# Association Between Anaemia and Dental Caries in Brazilian Adolescents

**DOI:** 10.3290/j.ohpd.b871067

**Published:** 2020-12-14

**Authors:** Isabelle Aguiar Prado, Cayara Mattos Costa, Susilena Arouche Costa, Cadidja Dayane Sousa do Carmo, Erika Bárbara Abreu Fonseca Thomaz, Soraia de Fátima Carvalho Souza, Cecilia Claudia Costa Ribeiro

**Affiliations:** a Dentist, Postgraduate Program of Dentistry, Federal University of Maranhão – Brazil. Contributed to data analysis and drafted the manuscript; read and approved the final article.; b Dentist, College of Dentistry, Federal University of Maranhão – Brazil. Contributed to data analysis and drafted the manuscript; read and approved the final article.; c Dentist, Postgraduate Program of Dentistry, Federal University of Maranhão – Brazil. Contributed to data analysis and drafted the manuscript; read and approved the final article.; d Professor, UNDB University Center, Brazil. Contributed to data acquisition and final review of the manuscript; read and approved the final article.; e Professor, Postgraduate Program of Public Health, Federal University of Maranhão – Brazil; Professor, Postgraduate Program of Dentistry, Federal University of Maranhão – Brazil. Contributed to conception, design, drafted and critically revised the manuscript; read and approved the final article.; f Professor, Postgraduate Program of Dentistry, Federal University of Maranhão – Brazil. Contributed to data analysis, critically revised the manuscript.; g Professor, Postgraduate Program of Public Health, Federal University of Maranhão – Brazil; Professor, Postgraduate Program of Dentistry, Federal University of Maranhão – Brazil. Contributed to conception, design, drafted and critically revised the manuscript; read and approved the final article.

**Keywords:** iron-deficiency anaemia, dental caries, adolescents

## Abstract

**Purpose:**

The present study analysed the association between anaemia and dental caries in adolescents on the basis of predisposing factors and presence of severely decayed teeth. Materials and Methods: This observational study included a complex probabilistic sample of adolescents (17–18 years old) enrolled at public schools in São Luís, Brazil (n = 363). Two hypothesis models were tested: (1) anaemia and dental caries are associated given that they share predisposing factors, such as socioeconomic and high sugar consumption, and (2) the presence of severely decayed teeth may increase the susceptibility of patients to anaemia. In the first model, the association between anaemia and the history of dental caries (the outcome number of affected teeth) was analysed by Poisson regression. In the second model, the association between the presence of severely decayed teeth with pulp exposure/necrosis and the outcome anaemia was analysed by logistic regression. Bivariate and multivariate analyses after adjusting for socioeconomic factors and sugar consumption were performed, considering 5% of statistical significance level and using STATA 115.0.

**Results:**

Anaemia was associated with a higher number of affected teeth with a history of dental caries in bivariate (means ratio [MR]: 1.30; 95% confidence intervals [CI95%]: 1.10–1.52; p = 0.001) and multivariate (MR: 1.18; CI95%: 1.01–1.39; p = 0.046) analyses. Severely decayed teeth with pulp exposure/necrosis were associated with anaemia in bivariate (odds ratios [OR]: 5.75; CI95%: 1.97–16.8; p = 0.001)] and multivariate (OR 5.51; CI95%: 1.71–17.74; p = 0.004) analyses.

**Conclusion:**

This study suggests that anaemia and dental caries are associated in a population-based sample of adolescents and that predisposing factors and severely decayed teeth seem to be involved in this association.

Nutritional anaemia occurs when the nutrient intake is insufficient to meet the demands of haemoglobin and erythrocyte synthesis.^26^ Iron deficiency is its most common cause.^19^,^26^ Adolescents are more vulnerable to anaemia due to physiological changes in this stage of life, which increases the iron demand in the body.^26^

Social vulnerability and unhealthy diets, notably high sugar consumption, would be the predisposing factors underlying the association between anaemia and dental caries. Individuals who have a lower socioeconomic level are the most exposed to high-sugar foods due to their low cost.^6^ A diet rich in sugar and poor in micronutrients can result in iron-deficiency anaemia^11^ and has been clearly implicated in dental caries.^5^,^23^,^28^

In an opposite direction of association, dental caries has been indicated as a risk factor for iron deficiency and anaemia in early childhood (<6 years of age).^8^ The infection from untreated cavities could be an explanation for the association between severely decayed teeth and anaemia.^8^ Two mechanisms may support this premise. Firstly, tooth pain during chewing limits the intake of fibrous foods and other iron-containing foods, which could result in anaemia.^13^ Secondly, the presence of infection/inflammation originating from the severely decayed teeth can interfere with erythropoiesis and reduce haemoglobin and iron levels in the blood.^2^

Assuming that adolescents are the largest consumers of added sugars,^3^,^9^ anaemia is highly prevalent in adolescents^21^ and that dental caries still remains a public health problem,^28^ we hypothesise an association between anaemia and dental caries in adolescents in a population-based sample. Therefore, the present study aimed to analyse the relationship between anaemia and dental caries in adolescents by testing two hypothesis models: (1) anaemia and dental caries are associated because they share predisposing factors; and (2) the presence of severely decayed teeth could increase the susceptibility of patients to anaemia.

## MATERIALS AND METHODS

### Study Design and Sample Selection

**Fig 1:**
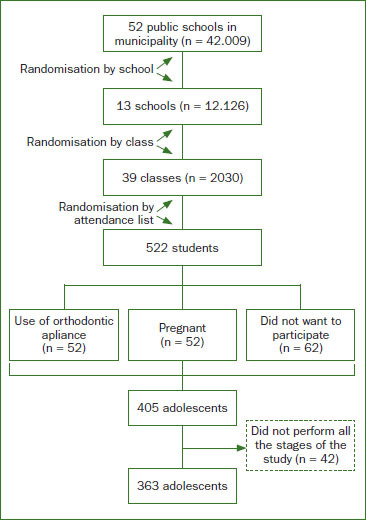
A three-stage complex probabilistic sample (random school, class, and student) was used.

This transversal study was performed from January 2014 to July 2016, as per the guidelines provided by Strengthening the Reporting of Observational Studies in Epidemiology for observational studies.^20^ The population-based sample comprised students (17–18 years of age) from public schools in São Luís, Brazil. A three-stage complex probabilistic sample: random school, class, and student was used (Fig 1). A description of the complex sampling methodology has been described in another study by this group.^4^

A sample of 294 students had 80% power to detect statistically significant odds ratios (OR) between the presence of anaemia and dental caries, considering ratio 1:1 between exposed and unexposed, prevalence of 27% in outcome among unexposed^14^ and 95% confidence level. To compensate for possible losses and considering the effect of sample design complex (deff = 1.1), an excess of 20% was added to the original number of participants calculated, resulting in a minimum sample of 354 students.

The study was approved by the Research Ethics Committee of the Federal University of Maranhão (number: 441.226). All the participants in the study and their legal representative agreed and signed a free informed consent form.

### Data Collection and Variables Analysis

The variables such as self-reported race/ethnicity (white, mulatto, and black), sex (male or female), and family income were collected using a structured questionnaire. The family income was collected in Brazilian minimum wage (approximately U$390.00 in August 2014) and it was divided into three categories: ≤ 1 minimum wage; between 1 and 2 minimum wage; and > 2 minimum wage.

Added sugar consumption was obtained from the application of a food frequency questionnaire validated for Brazilian adolescents.^7^ The measurement of added sugar consumption considered the summation of the intake of soft drinks, candies, cookies and chocolates. The variable was categorised according to the frequency: rarely or never; <2 days/week; 2–3 days/week; and 4–7 days/week.

Fasting blood samples were obtained from these adolescents to analyse serum haemoglobin concentration using the Sysmex XE-2100 equipment. Anaemia was defined according to World Health Organization (2011)^24^ for this age group: (a) female sex; Hb <12 g/dl and (b) male sex; Hb <13 g/dl.

The history of dental caries (past and current) was evaluated using the DMFT index (number of decayed, missing, and filled permanent teeth)^27^ and was used as a discrete variable. Severely decayed teeth were evaluated using the PUFA index (number of teeth with pulpal involvement, ulceration caused by dislocated tooth fragments, fistula and dentoalveolar abscess).^12^ The variable was categorised according to the number of severely decayed teeth: no teeth with pulpal involvement (PUFA = 0); one tooth with pulpal involvement (PUFA = 1); two or more teeth with pulpal involvement (PUFA ≥ 2). Data collection details and calibration of the evaluators have been published in another study by this group.^4^

### Study Models and Statistical Analysis

First, a model of association between the presence of anaemia and the history of dental caries in adolescents (number of DMFT) after adjusting for predisposing factors was tested. Bivariate analysis and multivariate analysis (adjusted for socioeconomic factors: skin colour, income, and sex and added sugar consumption frequency) were analysed by Poisson regression, thus estimating the means ratio (MR) and 95% confidence intervals (CI95%).

Then, the second model of association between anaemia and presence of severely decayed teeth was evaluated. Bivariate and multivariate analyses adjusted for confounders (socioeconomic factors and sugar consumption) were analysed by logistic regression, thus estimating OR and CI95%.

All analyses were performed using STATA 15.0 (Stata, College Station, TX, USA), with a 5% statistical significance level.

## RESULTS

A total of 42 adolescents (10.4%) did not complete all the stages of the investigation. Therefore, 363 individuals were included in this study. The sample comprised a majority of girls (55.65%); self-declared as mulatto (66.6%); and family income of one minimum Brazilian wage (50.41%). The prevalence of anaemia was 10.14%. The mean number of teeth with a history of dental caries (DMFT ≥ 1) was 3.72 (± 3.40). Severely decayed teeth (PUFA = 1 or PUFA ≥ 2) were present in 17.36% of the adolescents (Table 1).

**Table 1:** Descriptive characteristics of the adolescents examined, São Luís, Brazil

Variables	Mean	SD	n	%
Sex				
Male	–	–	161	44.35
Female	–	–	202	55.65
Self-declared skin colour				
Black	–	–	66	18.18
White	–	–	55	15.15
Mullato	–	–	242	66.67
Income				
≤ 1 MW	–	–	183	50.41
Between 1 and 2 MW	–	–	100	27.55
2 MW	–	–	80	22.04
Frequency of added sugar consumption				
Rarely or never	–	–	54	14.88
<2 days per week	–	–	114	31.40
2–3 days per week	–	–	125	34.44
4–7 days per week	–	–	70	19.28
Anaemia				
Yes	–	–	37	10.14
No	–	–	328	89.86
Severely decayed teeth (PUFA index)				
No tooth	–	–	300	82.64
One tooth	–	–	46	12.67
Two or more teeth	–	–	17	4.68
History of caries (DMFT index)	3.72	± 3.40	–	–

MW, minimum wage; PUFA, number of teeth with pulpal involvement, ulceration caused by dislocated tooth fragments, fistula and dentoalveolar abscess; DMFT, number of decayed, missing and filled teeth.

In the first model, anaemia was associated with higher numbers of teeth with a history of dental caries in bivariate (MR: 1.30; CI95%: 1.10–1.52; p = 0.001) and multivariate analyses (MR: 1.18; CI95%: 1.01–1.39; p = 0.046) (Table 2).

**Table 2:** Model of association between anaemia and history of caries in adolescents, São Luís, Brazil

	Bivariate analysis	Multivariate analysis^a^
	MR (CI95%) p value	MR (CI95%) p value
Anaemia associated to caries (number of teeth affected)	1.30 (1.10–1.52) p = 0.001	1.18 (1.01–1.39) p = 0.046

^a^ Adjusted for skin colour, family income, sex, frequency of sugar consumption; MR, mean ratio; CI95%, confidence interval of 95%.

In the second model, having two or more severely decayed teeth (PUFA ≥ 2) was the most strong exposure associated with anaemia in bivariate (OR: 5.75; CI95%: 1.97–16.8; p = 0.001) and multivariate analyses (OR: 5.51; CI95%: 1.71–17.74; p = 0.004) (Table 3).

**Table 3:** Model of association between severely decayed teeth and anaemia in adolescents, São Luís, Brazil

	Bivariate analysis	Multivariate analysis^a^
	OR (CI95%) p value	OR (CI95%) p value
Severely decayed teeth (PUFA index)		
No teeth	Reference	Reference
One tooth	1.28 (0.47–3.53) p = 0.63	0.92 (0.32–2.66) p = 0.87
Two or more teeth	5.75 (1.97–16.8) p = 0.001	5.51 (1.71–17.74) p = 0.004

^a^ Adjusted for skin colour, family income, sex, frequency of sugar consumption; OR, odds ratio; CI95%, confidence interval of 95%.

## DISCUSSION

The results of this population-level study confirm the hypothesis that anaemia and dental caries are associated in adolescents. We believe that predisposing factors (socioeconomic conditions and higher sugar consumption) and/or severely decayed teeth with pulp exposure/necrosis are the reasons underlying this association.

In the first model, a reduction in the strength of association between anaemia and the history of dental caries was observed while comparing the results of bivariate (MR: 1.30; p = 0.001) and multivariate analyses (MR: 1.18; p = 0.046). Therefore, the effect of model adjustment for socioeconomic conditions appears to be the predisposing factor underlying the association between anaemia and dental caries. Previous studies have already indicated socioeconomic conditions as causes of nutritional anaemia^1^ and dental caries;^15^ however, these studies evaluated these conditions separately.

Another explanation for the reduction in the strength of association between anaemia and dental caries in the first model can be the adjustment of the higher sugar consumption. Sugar plays a pivotal role in causing dental caries^17^,^23^ and its consumption appears to interfere in micronutrients deficiency, including iron.^10^ Adolescents have been increasingly exposed to the aggressive commercialisation of unhealthy food through social media. They are more susceptible to anaemia due to the lack of maturity, limited ability of cooking, and lack of resources/money for buying healthy foods.^22^ Therefore, youths consume relatively more ultraprocessed and higher sugar-added food and less fruits and vegetables.^22^,^25^ Furthermore, excessive sugar consumption can also interfere in the absorption and metabolism of minerals such as iron, even after appropriate supplementation.^18^

Socioeconomic conditions and high sugar consumption would not be the only explanations underlying the association between anaemia and dental caries in this study. In the first model, the strength of the association was reduced but remained statistically significant after adjustment for those predisposing factors (multivariate analysis), suggesting that other mechanisms are also involved in this process.

In the second model, two or more severely decayed teeth with pulp exposure/necrosis (PUFA≥2) were strongly associated with anaemia in the adolescents in bivariate and multivariate analyses. Pain and difficulty in chewing originated from the severely decayed teeth can compromise the intake and absorption of micronutrients such as iron.^2^,^14^ Moreover, infections resulting in the presence of inflammation in the severely decayed teeth can affect metabolic pathways and erythropoiesis, thus reducing the serum haemoglobin levels and anaemia.^16^,^29^

Our data support previous findings of association between dental caries and anaemia in early childhood shown by a systematic review, which included 15 studies using convenience samples, as hospitalised children or outpatients of paediatric hospitals.^8^ Our data contribute to the theme ‘anaemia and dental caries’ because we believe that this is the first population-based study to show the association between these two conditions in adolescents, including adjustment models for predisposing factors.

The only limitation of this study was its cross-sectional design. Data collection for anaemia and dental caries was made in the same time, which precludes the presumption of temporal relation between the variables to confirm a causal relationship. However, our data highlight that dental caries and anaemia coexist in adolescents.

One of the strengths of this study is the complex probabilistic sample of students enrolled at public schools in São Luís, Brazil. The other advantage of this study is that the statistical analysis was adjusted for predisposing factors to better understand the mechanisms that would be involved in the association between anaemia and dental caries.

## CONCLUSION

Our results indicate the association between anaemia and dental caries and their predisposing factors in adolescence. Hence, in adolescents with dental caries, a comprehensive health evaluation approach should be proposed, considering the higher susceptibility of these adolescents to anaemia due to social vulnerability and high sugar consumption.
